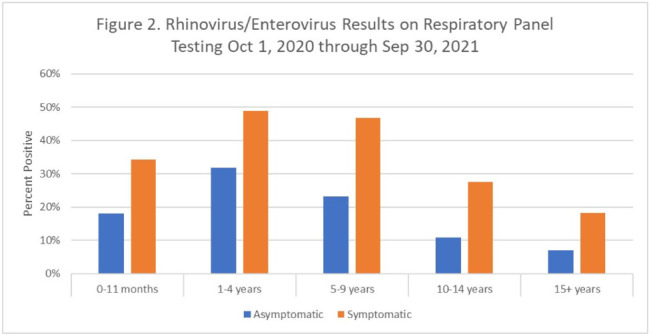# Respiratory Virus infections in symptomatic and asymptomatic children: Results of one year of hospital admission screening

**DOI:** 10.1017/ash.2022.175

**Published:** 2022-05-16

**Authors:** Zachary Most, Michael Sebert, Trish Perl

## Abstract

**Background:** Respiratory viral infections are very common among children. Transmission-based precautions are frequently used with patients who test positive for a respiratory virus in pediatric hospitals to prevent transmission of infections, regardless of whether the patient has symptoms of a respiratory infection or not (asymptomatic). However, few data are available on the prevalence of respiratory viral infections in symptomatic and asymptomatic children who are admitted to a pediatric hospital. The study was conducted in 3 hospitals that combine for a 601-bed pediatric healthcare system in northern Texas. **Methods:** From July 7, 2020, to the present, all patients admitted to the hospital had a nasopharyngeal swab collected and tested with a multiplex PCR panel including SARS-CoV-2 and 8 other common respiratory viruses. Over a 1-year period from October 1, 2020, to September 30, 2021, the prevalences of infection with each of the 9 respiratory viruses were calculated and stratified by respiratory infection symptom status (determined by the ordering provider in an electronic order set) and age group. **Results:** During this 1-year period, 28,421 PCR panels were collected on patients admitted to the hospital. The median age was 5 years (IQR, 1–12 years), and 15,105 patients were male (53.2%). Overall, 12,792 panels were positive for at least 1 virus (45.0%). Among 26,688 panels on individuals with known symptom status, 26.3% of asymptomatic patients and 69.4% of symptomatic patients tested positive for at least 1 virus. The most common virus was rhinovirus or enterovirus (17.7% asymptomatic positive and 40.2% symptomatic positive) (Fig. 1). Asymptomatic rhinovirus or enterovirus prevalence varied by age group and was greatest in children aged 1–4 years (31.7%) and those aged 5–9 years (23.1%). It was lowest in adolescents aged 15–21 years (7.1%) (Fig. 2). Over time, the prevalence of asymptomatic infections fluctuated with local outbreaks. For SARS-CoV-2, in the resolution phase of an outbreak the prevalence of asymptomatic infections tended to overlap or surpass symptomatic infections. **Conclusions:** Asymptomatic respiratory viral infections, and in particular rhinovirus or enterovirus infections, were common among pediatric patients admitted to the hospital during the COVID-19 pandemic and were most common among children aged 1–9 years. However, symptomatic patients were still more likely to test positive for a respiratory virus compared to asymptomatic patients. Prolonged shedding of SARS-CoV-2 may explain why asymptomatic prevalence surpasses symptomatic prevalence in the resolution phase after outbreaks.

**Funding:** None

**Disclosures:** None

**Figure:**
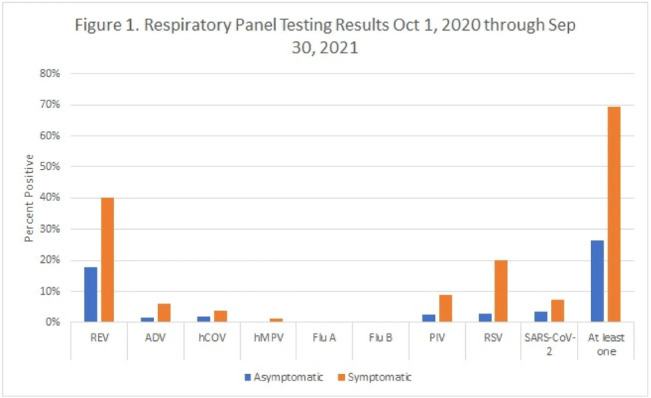

**Figure:**